# Which Definition of Upper Rectal Cancer Is Optimal in Selecting Stage II or III Rectal Cancer Patients to Avoid Postoperative Adjuvant Radiation?

**DOI:** 10.3389/fonc.2020.625459

**Published:** 2021-02-12

**Authors:** Xian Hua Gao, Bai Zhi Zhai, Juan Li, Jean Luc Tshibangu Kabemba, Hai Feng Gong, Chen Guang Bai, Ming Lu Liu, Shao Ting Zhang, Fu Shen, Lian Jie Liu, Wei Zhang

**Affiliations:** ^1^Department of Colorectal Surgery, Changhai Hospital, Shanghai, China; ^2^Hereditary Colorectal Cancer Center and Genetic Block Center of Familial Cancer, Changhai Hospital, Shanghai, China; ^3^Department of Colorectal Surgery, The 6th People’s Hospital of Kunshan, Suzhou, China; ^4^Department of Nephrology, Changhai Hospital, Shanghai, China; ^5^Department of General Surgery, Central Military Hospital, Kinshasa, Democratic Republic of Congo; ^6^Department of Pathology, Changhai Hospital, Shanghai, China; ^7^Department of Radiology, Changhai Hospital, Shanghai, China

**Keywords:** upper rectal cancer, anterior peritoneal reflection, intraoperative finding, MRI, radiotherapy

## Abstract

**Background:**

In most guidelines, upper rectal cancers (URC) are not recommended to take neoadjuvant or adjuvant radiation. However, the definitions of URC vary greatly. Five definitions had been commonly used to define URC: 1) >10 cm from the anal verge by MRI; 2) >12 cm from the anal verge by MRI; 3) >10 cm from the anal verge by colonoscopy; 4) >12 cm from the anal verge by colonoscopy; 5) above the anterior peritoneal reflection (APR). We hypothesized that the fifth definition is optimal to identify patients with rectal cancer to avoid adjuvant radiation.

**Methods:**

The data of stage II/III rectal cancer patients who underwent radical surgery without preoperative chemoradiotherapy were retrospectively reviewed. The height of the APR was measured, and compared with the tumor height measured by digital rectal examination (DRE), MRI and colonoscopy. The five definitions were compared in terms of prediction of local recurrence, survival, and percentages of patients requiring radiation.

**Results:**

A total of 576 patients were included, with the intraoperative location of 222 and 354 tumors being above and straddle/below the APR, respectively. The median distance of the APR from anal verge (height of APR) as measured by MRI was 8.7 (range: 4.5–14.3) cm. The height of APR positively correlated with body height (r=0.862, P<0.001). The accuracy of the MRI in determining the tumor location with respect to the APR was 92.1%. Rectal cancer above the APR had a significantly lower incidence of local recurrence than those straddle/below the APR (P=0.042). For those above the APR, there was no significant difference in local recurrence between the radiation and no-radiation group. Multivariate analyses showed that tumor location regarding APR was an independent risk factor for LRFS. Tumor height as measured by DRE, MRI and colonoscopy were not related with survival outcomes. Fewer rectal cancer patients required adjuvant radiation using the definition by the APR, compared with other four definitions based on a numerical tumor height measured by MRI and colonoscopy.

**Conclusions:**

The definition of URC as rectal tumor above the APR, might be the optimal definition to select patients with stage II/III rectal cancer to avoid postoperative adjuvant radiation.

## Introduction

Preoperative chemoradiotherapy (CRT) has become an integral part of the multimodal treatment for stage II and III rectal cancer. Preoperative CRT has been shown to improve the local control and sphincter preservation rates, without significant effect on the overall survival (OS) and disease-free survival (DFS) (1–3). However, the benefit of radiation for upper rectal cancer (URC) is not clear. The Dutch TME trial (4) and Swedish rectal cancer trial (5) demonstrated that, although local recurrence in the middle and lower rectum was significantly reduced by preoperative radiation, no significant reduction in local recurrence was found in patients with URC. Prior to the widespread use of total mesorectal excision (TME), postoperative adjuvant radiation was believed to potentially compensate for suboptimal surgical resection (6). However, with the advances in systemic chemotherapy and the quality of surgical excision, especially the increased use of TME, local recurrence of rectal cancer has decreased dramatically in the last three decades (6, 7). Moreover, considering the significant long-term side effects of radiation and a lack of clear benefit for URC, most current guidelines don’t recommend preoperative or postoperative radiation for URC (2, 3, 8–10). However, the definitions of URC vary greatly across these guidelines. In the 2020 NCCN guidelines, URC was defined as a rectal tumor with inferior margin located between the anterior peritoneal reflection (APR) and the sacral promontory, as determined by MRI (1). According to the 2017 ESMO guidelines, URC was defined as a tumor with inferior margin located at 10–15 cm from the anal margin, as measured by rigid sigmoidoscopy (2). In the German Guideline Program in Oncology (GGPO) 2019 guidelines, URC was defined as a tumor located at 12–16 cm from the anal verge as measured by rigid rectoscopy (9). In the Chinese Society of Clinical Oncology (CSCO) 2018 guidelines, URC was defined as a tumor located 10 cm above the anal verge, as observed on the MRI (10).

Although the tumor height-related definitions provide a reproducible method for defining URC, the body habitus and sex must be considered during the assessment of tumor location as, for instance, the rectum is longer in taller patients (3). The decision to administer radiation solely based on the numerical tumor height involves anatomical pitfalls. The distance between the anal margin and the APR varies from 3.5 to 16 cm, depending on the height, sex and age of the patient (11–14). Rectal cancers located 3.5–16 cm from the anal verge can also be intraperitoneal, which is often too large of a range to be reliably targeted with radiation; or it can be extraperitoneal, which should be amenable to receive radiation. Therefore, some surgeons propose that the APR could be a suitable landmark for identifying patients with rectal cancer for radiation (15). Furthermore, the 2020 NCCN guidelines also suggest that rectal tumor above the APR should be defined as URC (1). The overall reported 5-year local recurrence rate for intraperitoneal and extraperitoneal rectal cancer is 4.2% and 13.3%, respectively (15). There is also growing evidence that radiation may not be useful for intraperitoneal cancers (16–18). More importantly, blood-borne metastases or disseminated disease is predominant among intraperitoneal rectal tumors, whereas local failure is more frequent among extraperitoneal tumors. In addition, there is evidence that the rectum above the APR is quite distinct from that below the APR in terms of embryology, morphology, function and lymphatic drainage (11). The APR is a distinct anatomical landmark which could be easily identified by intraoperative examination and preoperative MRI (17, 19). Based on these reasons, we hypothesized that the definition of URC as a rectal tumor above the APR is the optimal definition to identify patients with stage II/III rectal cancer that should avoid radiation (11).

Five definitions of URC were used in this study and are as follows: 1) tumor >10 cm from the anal verge by MRI; 2) tumor >12 cm from the anal verge by MRI; 3) tumor >10 cm from the anal verge by flexible colonoscopy; 4) tumor >12 cm from the anal verge by flexible colonoscopy; 5) rectal tumor above the APR (1). In this study, we aimed to compare the five different definitions of URC for predicting OS, DFS and local recurrence free survival (LRFS), radiation effect and percentages of patients requiring radiation.

## Methods

### Patients

In this retrospective study, all consecutive patients with rectal cancer who underwent radical resection of the primary tumor between July 2017 and October 2018 at Changhai Hospital were included. This study was approved by the Ethics Committee of Changhai Hospital, which waived the requirement for informed consent as it was a retrospective study. The study was conducted in accordance with the principles of the Declaration of Helsinki. Perioperative clinicopathological parameters, tumor height, and tumor location relative to the APR were recorded and maintained in our colorectal cancer database.

### Selection Criteria

Inclusion criteria: 1) adult patients (> 18 years) of either sex with histopathologically confirmed rectal adenocarcinoma; 2) tumor within 15cm from the anal verge by flexible colonoscopy; 3) pathological stage II (T3-4N0M0) or stage III (T1-4N1-2 M0) rectal cancer; 4) underwent curative resection of primary rectal cancer; 5) without preoperative CRT.

Exclusion criteria: 1) patients who underwent palliative resection; 2) positive resection margin (including proximal, distal and circumferential); 3) synchronous or metachronous multiple primary colorectal cancer; 4) hereditary colorectal cancer syndrome; 5) previous history of pelvic radiation; 6) preoperative concomitant intestinal obstruction or perforation; 7) patients without recurrence who did not complete at least 24 months of follow-up after primary surgery.

### Surgery and Histopathological Assessment

All surgeries were performed by seven chief surgeons, each with the experience of performing at least 100 operations for colorectal cancer per year, following a standardized operation protocol (including standard TME and high vascular ligation of the inferior mesenteric artery and vein). All resected specimens were examined using a standardized protocol that included TNM classification according to the American Joint Committee on Cancer-International Union Against Cancer (8^th^ edition). Resection margins, including circumferential, proximal, and distal margins, were considered positive if tumor cells were identified within 1 mm of the surgical resection margin.

### Postoperative CRT

For middle and lower rectal cancer, preoperative CRT was recommended to all patients with stage II/III tumor, and some patients refused. For upper rectal cancer, preoperative CRT was not recommended to patients with stage II/III tumor (excluding T4b). All patients with stage II/III rectal cancer who did not receive preoperative CRT were recommended to undergo postoperative CRT, and some patients refused to take postoperative CRT or chemotherapy. Postoperative adjuvant CRT was initiated 4 weeks after surgery and continued for 6 months. The dose of postoperative adjuvant radiation was 1.8 to 2.0 Gy daily for a total of 23 to 28 fractions over 5–6 weeks and resulted in a total dose of 46.0 to 50.4 Gy. Postoperative adjuvant radiation was delivered by the three-field or four-field box technique to the original area of tumor and mesorectum, presacral region, and the internal iliac lymph nodes. Postoperative adjuvant concurrent chemotherapy [capecitabine (1000 mg/m^2^, twice daily)] was administered orally throughout the period of radiation treatment. Postoperative adjuvant chemotherapy (CapeOX or mFOLFOX6) was administered 3 weeks after the completion of radiation and continued for 4–6 months.

### Follow Up

Clinical follow-up consisted of physical examination, DRE, chest CT scan, liver contrast-enhanced MRI, rectal contrast-enhanced MRI and the serum level measurement of CEA and CA19-9. These examinations were performed every 3 months for the first 2 years after surgery, every 6 months for another 3 years and annually thereafter. A flexible colonoscopy was performed annually for 5 years. The local recurrence and distant metastasis were confirmed by biopsy when appropriate or based on the progressive increase in the size of the lesions or the appearance of new lesions.

### Definition of Parameters

*Rectal cancer* was defined as a large intestine tumor with its lower margin located within 15cm from the anal verge by flexible colonoscopy. *Local recurrence* was defined as evidence of recurrent disease within the pelvis after radical resection, including recurrence at the site of anastomosis, the pelvic cavity and the perineal wound. *LRFS* was defined as the period between the date of surgery for primary rectal tumor and the date of local recurrence, or death from any cause. *DFS* was defined as the time between the date of surgery for primary rectal tumor and the date of local recurrence, distant metastasis or death from any cause. ***OS*** was defined as the time interval between the date of surgery for primary rectal tumor and the date of death or last follow-up, with no restriction on the cause of death.

### Measurements of Tumor Height and Tumor Location Relative to the APR

**Figure 1:**
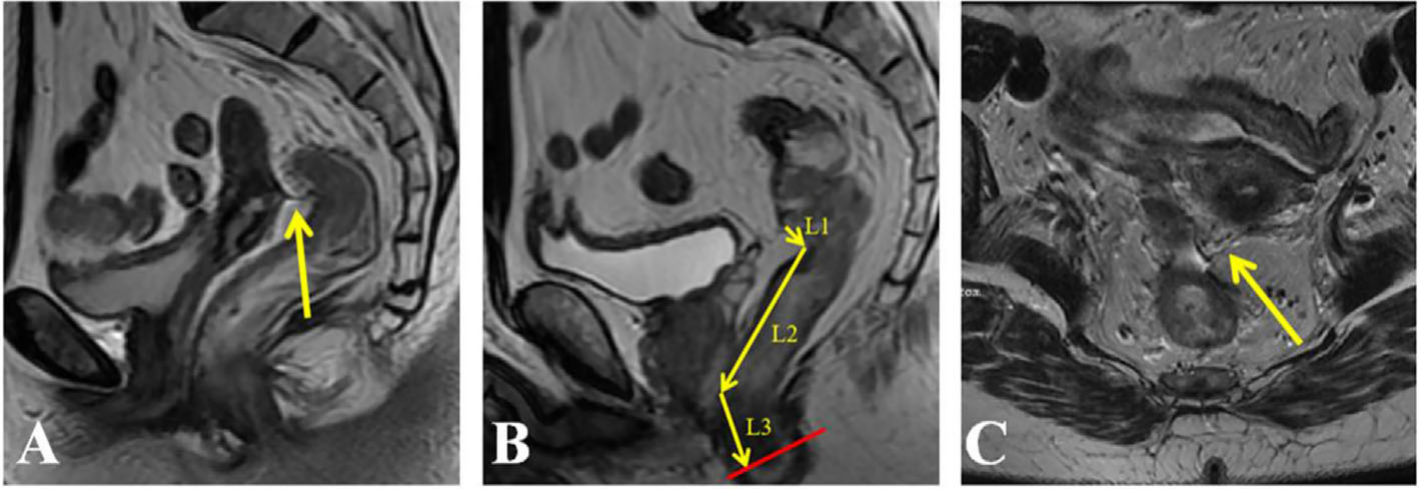
Identification of the anterior peritoneal reflection (APR) and measurement of tumor height on an MRI. **(A)** APR (arrow) in the sagittal plane; **(B)** the height of the tumor defined as the distance from the anal verge to the inferior tumor margin was measured in sagittal images; **(C)** APR (arrow) in the axial plane.

**Figure 2:**
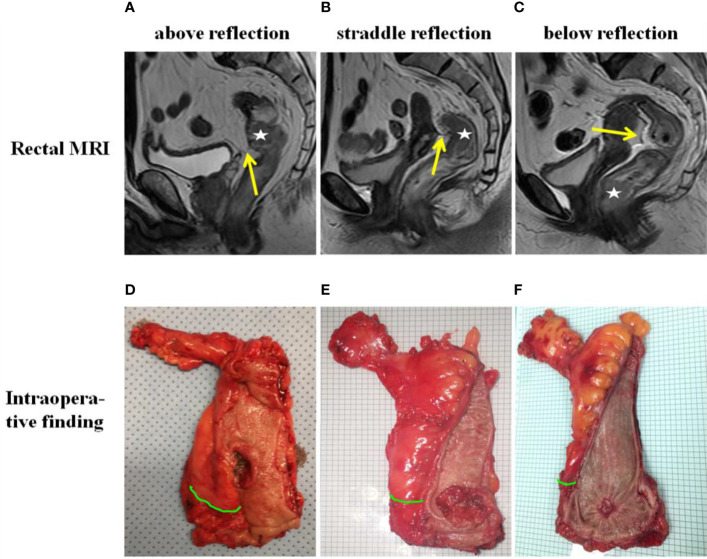
Tumor location relative to the anterior peritoneal reflection (APR) as determined by MRI **(A–C)** and intraoperative palpation and visualization **(D–F)**. The “☆” in the MRI indicates the tumor. The yellow arrow in the MRI indicates the APR. The green curve in intraoperative finding indicates the APR.

Preoperatively, tumor height was measured by DRE, flexible colonoscopy and rectal contrast-enhanced MRI. The relationship between the APR and inferior tumor margin was determined by preoperative MRI. Both DRE and flexible colonoscopy were performed by experienced colorectal surgeons. All rectal MRI images were reviewed by experienced radiologists on the PACS system. Sagittal and axial T2-weighted images were used for the identification of the APR. In the midsagittal plane, the APR was identified as a thin hypointense line extending from the superior aspect of the urinary bladder (men) or uterus (women) to the anterior rectal wall (**Figure 1A**). The height of the tumor was defined on the sagittal images as the distance from the anal verge to the inferior tumor margin (**Figure 1B**). In some cases, it was necessary to interconnect two or more angulated lines, sometimes on two or more adjacent sagittal slices for an approximate total length (13). On axial imaging, the APR attached to the anterior rectal wall in a V‐shaped hypointense configuration (**Figure 1C**). MRI was also used to identify the relationship between the tumor and the APR preoperatively (**Figures 2A–C**, **Supplemental Figures 1A–C**). The relationship between tumor location and the APR was also determined intraoperatively by palpation and visualization **Figures 2D–F**). All patients had an intraoperative assessment of the APR. Based on intraoperative findings, rectal cancer patients were classified into the “above the APR” group, or the “straddle/below the APR” group. The accuracy of the MRI in determining tumor location relative to the APR, was calculated using the intraoperative finding as the gold standard.

### Statistical Analysis

Statistical analysis was performed with the SPSS 22.0 software (Chicago, IL). The “t” test or Wilcoxon test was used to compare continuous variables. The Chi-square test or Fisher exact test was used to compare categorical variables. The Pearson correlation coefficient was used to characterize the agreement between the measurements by DRE, flexible colonoscopy, and MRI. The agreement between each pair of measurements was compared using the Bland and Altman plot. The Kaplan-Meier analysis and log-rank tests were used to compare survival differences between two groups. Multivariable Cox proportional hazard analyses were used to explore the factors affecting OS, DFS, and LRFS. All parameters which showed statistical significance in the univariate analysis or had potential clinical significance were included into the multivariate analysis. The multivariate Cox proportional hazard analysis was employed using the stepwise method (forward: likelihood ratio) with an entry criterion of P<0.05 and a removal criterion of P>0.10. For all analyses, P<0.05 (two-sided) was considered statistically significant.

## Results

### Patient Characteristics

**Figure 3:**
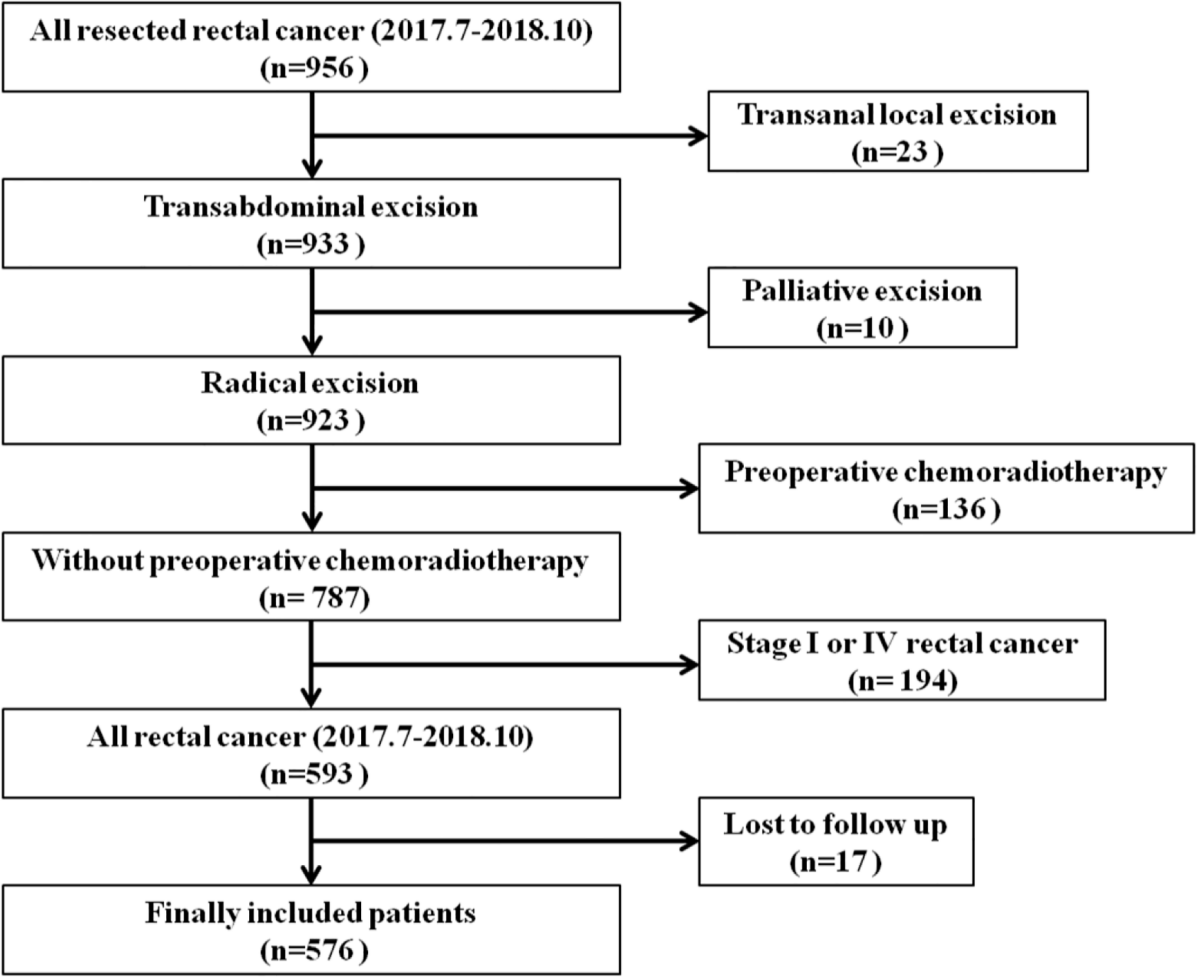
Flow chart of patient selection.

A total of 576 patients with rectal cancers were included in this study, with 222 tumors above the APR and 354 tumors straddle/below the APR as determined by intraoperative findings. The flowchart of patient selection is shown in **Figure 3**. The median age of patients was 63 years (interquartile range, 54 to 69 years), and the median follow up period was 22 months (interquartile range, 18 to 28 months). The demographic, clinicopathological and treatment data are presented in **Table 1**.

**Table 1:** The demographic, clinicopathological and treatment details of the study patients.

Parameters		Tumor location in relation to the APR		P value
		Above (n=222)	Straddle or below (n=354)	
Sex (Male/Female)		154/68	241/113	0.745
Age (year)		61.35±10.87	61.34±10.74	0.987
Body height (cm)		166.39±8.24	166.17±7.80	0.750
Body weight (kg)		66.74±11.90	64.70±10.65	**0.038**
BMI (kg/m^2^)		23.99±3.24	23.36±3.05	**0.019**
Tumor diameter (cm)*		4.17±1.40	4.18±1.45	0.969
Tumor height by DRE (cm)*		6(4–8); n=74	4(0–8); n=325	**<0.001**
Tumor height by colonoscopy (cm)*		10(4–15); n=215	4(0–15); n=346	**<0.001**
Tumor height by MRI (cm)*		9(4.4–15); n=127	5(0–13); n=257	**<0.001**
Preoperative T stage	1	6(33.3%)	12(66.7%)	0.610
	2	59(38.3%)	95(61.7%)	
	3	147(38.2%)	238(61.8%)	
	4	10(52.6%)	9(47.4%)	
Preoperative N stage	0	97(40.6%)	142(59.4%)	0.587
	1	83(38.2%)	134(61.8%)	
	2	42(35.0%)	78(65.0%)	
Preoperative TNM stage	I	21(43.8%)	27(56.3%)	0.614
	II	76(39.8%)	115(60.2%)	
	III	125(37.1%)	212(62.9%)	
Postoperative pathological TNM stage	II	107(42.5%)	145(57.5%)	0.088
	III	115(35.5%)	209(64.5%)	
Differentiation	Well	2(40.0%)	3(60.0%)	0.624
	Moderate	196(39.3%)	303(60.7%)	
	Poor	24(33.3%)	48(66.7%)	
Tumor deposit	No	186(38.8%)	294(61.3%)	0.818
	Yes	36(37.5%)	60(62.5%)	
Lymphovascular invasion	No	181(40.0%)	272(60.0%)	0.181
	Yes	41(33.3%)	82(66.7%)	
Perineural invasion	No	159(38.7%)	252(61.3%)	0.910
	Yes	63(38.2%)	102(61.8%)	
Tumor budding	No	164(37.4%)	275(62.6%)	0.296
	Yes	58(42.3%)	79(57.7%)	
dMMR status	pMMR	210(38.2%)	340(61.8%)	0.414
	dMMR	12(46.2%)	14(53.8%)	
KRAS	Wild type	131(40.1%)	196(59.9%)	0.391
	Mutant type	91(36.5%)	158(63.5%)	
NRAS	Wild type	211(38.2%)	341(61.8%)	0.453
	Mutant type	11(45.8%)	13(54.2%)	
BRAF	Wild type	217(38.5%)	347(61.5%)	0.822
	Mutant type	5(41.7%)	7(58.3%)	
Postoperative radiation	Yes	186(43.3%)	244(56.7%)	**<0.001**
	No	36(24.7%)	110(75.3%)	
Postoperative chemotherapy	Yes	37(34.3%)	71(65.7%)	0.310
	No	185(39.5%)	283(60.5%)	
CEA	<5 ng/ml	146(37.9%)	239(62.1%)	0.664
	>= 5 ng/ml	76(39.8%)	115(60.2%)	
CA199	< 37 U/ml	198(38.6%)	315(61.4%)	0.939
	>= 37 U/ml	24(38.1%)	39(61.9%)	

*Median (range). BMI, body mass index; DRE, digital rectal examination; APR, anterior peritoneal reflection; dMMR, deficient mismatch repair; pMMR, proficient mismatch repair. In bold: p < 0.05.

Of the included 576 patients with rectal cancer, 384 had preoperative rectal MRI imaging and 102 patients had only preoperative rectal CT scan in our picture archiving and communication (PACS) system. The remaining 90 had preoperative assessment at other hospitals. The APR was visible in 330 cases (85.9%) on the rectal MRI. The median distance between the APR and the anal verge was 8.7 (range: 4.5–14.3) cm (**Supplemental Table 1**). The median distance between the APR and the anal verge was significantly higher in the males compared to females [8.9 (range: 5.3–14.3) cm vs. 8.4 (range: 4.5–12.9) cm, P = 0.001]. The distance of the APR from the anal verge showed a positive correlation with body height (r = 0.862, P < 0.001), and could be calculated with the following formula: distance (cm) = [0.1 × height (cm)] - 8.0. The accuracy of the MRI in determining tumor location relative to the APR was 92.1%. The accuracy of MRI to identify the tumors above, straddle and below the APR was 89.1%, 95.3%, and 93.1%, respectively (**Supplemental Table 2**). The Kappa value of tumor location with respect to the APR, as determined by MRI and intraoperative findings, was 0.881 (P < 0.001).

### The Relationship Between Tumor Location Relative to the APR, Postoperative Radiation and Survival Related Parameters (OS, DFS, and LRFS)

During the follow-up period, a total of 39 deaths occurred, including 32 (82.1%) from rectal cancer, 5 (12.8%) from cardiovascular diseases and 2 (5.1%) from unknown causes. Eight patients (1.4%) developed local recurrence [1 (0.5%) patient with tumor above the APR and 7 (2.0%) patients with tumor straddle/below the APR]. Local recurrence and distant metastasis occurred in 1.4% and 12.0% of patients at 2 years, respectively. The actual 2-year rate of OS, DFS and LRFS were 95.0%, 86.8% and 91.5%, respectively.

**Figure 4:**
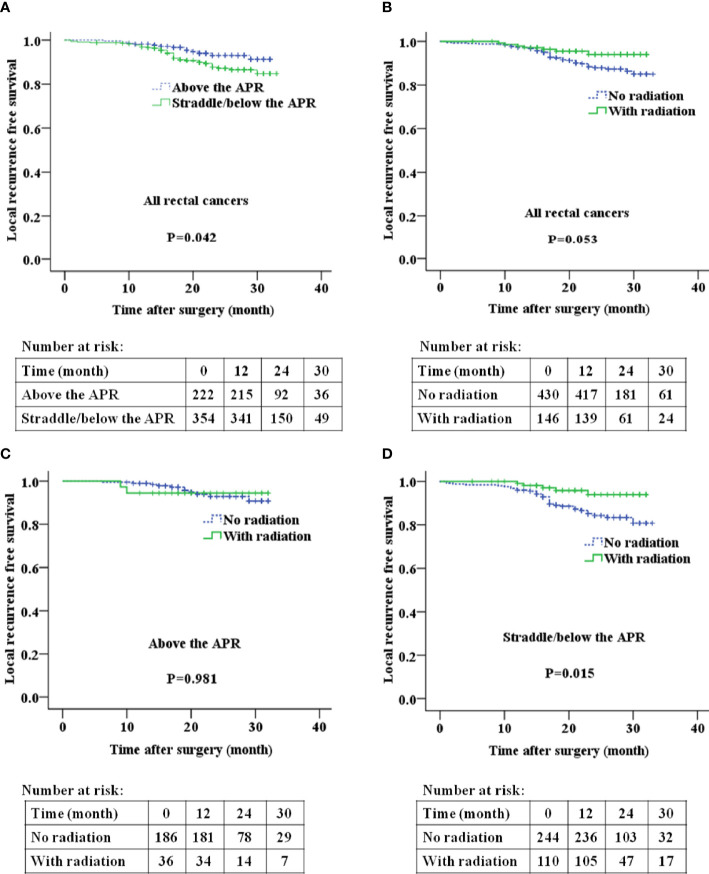
The relationship between tumor location relative to the APR and local recurrence free survival **(A, B)**, postoperative radiation and local recurrence free survival **(C, D)** in patients with rectal cancer. APR: anterior peritoneal reflection.

Rectal cancer above the APR exhibited a significantly lower incidence of local recurrence than those straddle/below the APR (**P=0.042**, **Figure 4A**). No significant difference was identified for OS and DFS between the two groups (**Supplemental Figures 2A** and **3A**). No significant difference was identified for OS (**Supplemental Figure 2B**), DFS (**Supplemental Figure 3B**) and LRFS (**Figure 4B**) between the radiation group and the no-radiation group. Subgroup analyses revealed that, for patients with rectal cancer above the APR, there was no significant difference in LRFS between the radiation group and the no-radiation group (**Figure 4C**). For patients with rectal cancer straddle/below the APR, the radiation group had significant longer LRFS than the no-radiation group (**Figure 4D**).

### Univariate and Multivariate Analyses of Risk Factors Affecting OS, DFS, and LRFS

Univariate and multivariate analyses demonstrated that the degree of tumor differentiation, tumor deposits, lymphovascular invasion and perineural invasion were independent risk factors affecting OS (**Supplemental Table 3**). Postoperative pathological TNM stage, tumor deposit and lymphovascular invasion were independent risk factors affecting DFS (**Supplemental Table 3**). Postoperative pathological TNM stage, differentiation, tumor deposit, perineural invasion, tumor budding, postoperative radiation [0.20(0.08-0.46), P < 0.001], postoperative chemotherapy [0.44(0.24–0.81), P = 0.008] and tumor location with regards to the APR [1.97(1.04–3.72), P=0.038] were independent predictors of LRFS (**Table 2**).

**Table 2:** Univariate and multivariate analyses of risk factors of LRFS using a Cox regression model (n = 576).

Parameters	LRFS			
	Univariate		Multivariate	
	HR(95%CI)	P	HR(95%CI)	P
Gender (male vs. female)	0.96(0.53–1.73)	0.894		
BMI (>=23.59 vs. <23.59kg/m^2^)	1.05(0.61–1.82)	0.850		
Diameter (>=4 vs. <4cm)	1.09(0.62–1.92)	0.756		
Postoperative pathological TNM stage (III vs. II)	3.10(1.60–6.04)	**0.001**	2.38(1.13–5.04)	**0.023**
Differentiation (Poor vs. Well/moderate)	2.29(1.17–4.46)	**0.015**	2.39(1.19–4.78)	**0.014**
Tumor deposit (Yes vs. No)	3.47(2.00–6.05)	**<0.001**	2.70(1.45–5.02)	**0.002**
Lymphovascular invasion (Yes vs. No)	2.43(1.39–4.25)	**0.002**	1.18(0.62–2.24)	0.608
Perineural invasion (Yes vs. No)	3.39(1.96–5.86)	**<0.001**	2.72(1.55–4.79)	**0.001**
Tumor budding (Yes vs. No)	2.59(1.44–4.66)	**0.002**	2.62(1.45–4.76)	**0.002**
dMMR status (dMMR vs. pMMR)	0.46(0.06–3.32)	0.440		
KRAS (Mutant vs. Wild)	1.43(0.83–2.46)	0.198		
NRAS (Mutant vs. Wild)	0.05(0.00–28.92)	0.351		
BRAF (Mutant vs. Wild)	0.05(0–1697.75)	0.571		
Postoperative radiation (Yes vs. No)	0.47(0.21–1.03)	**0.060**	0.20(0.08–0.46)	**<0.001**
Postoperative chemotherapy (Yes vs. No)	0.41(0.23–0.72)	**0.002**	0.44(0.24–0.81)	**0.008**
CEA (>= 5 vs. <5 ng/ml)	1.45(0.84–2.52)	0.184		
CA19-9 (>= 37 vs. <37U/ml)	2.44(1.26–4.75)	**0.009**	1.79(0.88–3.65)	0.107
**Tumor location relative to the APR (straddle/below vs. above)**	1.89(1.01–3.55)	**0.046**	1.97(1.04–3.72)	**0.038**

BMI, body mass index; APR, anterior peritoneal reflection; dMMR, deficient mismatch repair; pMMR, proficient mismatch repair. In bold: p < 0.05.

### Consistency of Tumor Height Measured by DRE, MRI, and Flexible Colonoscopy

All patients underwent DRE and the inferior tumor margin could be reached by an examiner’s finger in 399 cases. In addition, 576 patients had undergone a flexible colonoscopy, and 384 received rectal contrast MRI preoperatively. The tumor height in 290 cases, as measured by DRE and MRI, were correlated with each other (Pearson correlation coefficient R = 0.723, **Supplemental Figure 4A**), as indicated by the regression equation (*y* = 0.79*x* + 1.93). The tumor height measured by DRE was correlated with that measured by colonoscopy in 394 cases (R = 0.785, *y* = 1.11*x* -0.30, **Supplemental Figure 4B**). The tumor height measured by colonoscopy was correlated with that measured by MRI in 377 cases (R = 0.822, *y* = 0.67*x* + 2.58, **Supplemental Figure 4C**).

To estimate the degree of measurement difference in each individual, we used the Bland and Altman plot. In the scatter plot between DRE and MRI (**Supplemental Figure 4D**), the mean difference was –1.1 cm (95% CI: –4.2 to 2.1 cm). Similarly, the mean difference between DRE and colonoscopy was –0.2 cm (95% CI: –3.7 to 3.3 cm) (**Supplemental Figure 4E**), and the mean difference between colonoscopy and MRI was –0.7 cm (95% CI: –4.8 to 3.4 cm) (**Supplemental Figure 4F**).

### The Relationship Between Survival Related Parameters and Tumor Height as Measured by DRE, MRI, and Flexible Colonoscopy

Kaplan-Meier analyses found no significant difference in OS, DFS, or LRFS between these two groups divided by a fixed tumor height [colonoscopy and MRI (> 10 vs. <= 10 cm, > 12 vs. <= 12 cm)] (**Table 3**). Patients with rectal cancer above the APR had significantly longer LRFS than those straddle/below the APR (P = 0.046), but not for OS or DFS (**Table 3**).

**Table 3:** Kaplan-Meier analysis of the relationship between tumor height-related parameters and survival outcomes.

Parameters	OS		DFS		LRFS	
	HR(95%CI)	P	HR(95%CI)	P	HR(95%CI)	P
Tumor height by MRI (<=10 vs. >10 cm)	0.91(0.26–3.16)	0.887	0.67(0.30–1.48)	0.324	0.41(0.13–1.35)	0.143
Tumor height by MRI (<=12 vs. >12 cm)	1.41(0.32–6.16)	0.648	0.21(0.03–1.53)	0.124	0.33(0.05–2.41)	0.274
Tumor height by colonoscopy (<=10 vs. >10 cm)	1.54(0.76–3.14)	0.230	1.35(0.86–2.10)	0.187	1.05(0.57–1.94)	0.880
Tumor height by colonoscopy (<=12 vs. >12 cm)	2.02(0.87–4.65)	0.100	1.58(0.91–2.76)	0.106	1.91(0.96–3.83)	0.067
**Tumor location in relation to the APR (straddle/below vs. above)**	1.01(0.50–2.01)	0.983	1.20(0.78–1.85)	0.416	**1.89(1.01–3.55)**	**0.046**

In bold: p < 0.05.

### Different Percentages of rectal Cancer Patients Requiring Radiation Based on the Five Commonly Used Definitions of URC

Most guidelines do not recommend patients with stage II/III URC to receive neoadjuvant or adjuvant radiation. However, the definition of URC varied greatly in different guidelines. Here we compared the percentages of rectal cancer patients requiring radiation based on the five commonly used definitions of URC. The results demonstrated that fewer patients required radiation using the definition based on the APR (61.5%) compared with the other four definitions using a numerical tumor height measured by MRI and colonoscopy (64.2%–100.0%, **Table 4**).

**Table 4:** The percentages of rectal cancer patients requiring postoperative radiation based on 5 commonly used definitions of URC.

No.	Definitions of URC	Requiring postoperative radiation		Total
		Yes	No	
1	>10 cm from the anal verge by MRI	319(83.1%)	65(16.9%)	384
2	>12 cm from the anal verge by MRI	357(93.0%)	27(7.0%)	384
3	>10 cm from the anal verge by colonoscopy	410(71.2%)	166(28.8%)	576
4	>12 cm from the anal verge by colonoscopy	496(86.1%)	80(13.9%)	576
5	Above the APR	354(61.5%)	222(38.5%)	576

URC, upper rectal cancer; APR, anterior peritoneal reflection.

## Discussion

Although most current guidelines do not recommend radiation for URC, the definitions of URC vary greatly across these different guidelines. The present study showed that the height of the APR, which correlates with sex and body height, is a distinct and individualized landmark. Rectal cancer above the APR had a significantly lower incidence of local recurrence than those that straddle/below the APR. Univariate and multivariate COX analyses demonstrated that tumor location relative to the APR was an independent risk factor of LRFS, while other tumor height related parameters measured by DRE, colonoscopy and MRI were not related to OS, DFS, or LRFS. Subgroup analyses showed that, only in patients with rectal cancer straddle/below the APR, the radiation group had significant longer LRFS than the no-radiation group. Moreover, fewer rectal cancer patients required radiation when URC was defined by the APR compared with those defined by the other four definitions. Hence, we suggest that the definition of URC as a rectal tumor above the APR might be the optimal definition in selecting patients with stage II/III rectal cancer to avoid radiation.

To the best of our knowledge, this is the first study focusing on the definition of URC in identifying patients with stage II/III rectal cancer that should avoid radiation. The 2020 NCCN guidelines defined URC as a rectal tumor above the APR, but it recommended that all patients with URC should receive neoadjuvant or adjuvant radiation (1). In this study, we found that the optimal definition of URC was rectal tumors above the APR, and for these patients there was no significant difference between the radiation group and the no-radiation group in terms of OS, DFS and LRFS. The tumor location relative to the APR can be determined by intraoperative findings and preoperative rectal MRI, and the APR can be easily identified during open or laparoscopic surgery (12, 14). For the selection of postoperative radiation, intraoperative determination of the tumor location relative to the APR is more direct and accurate. When it comes to the selection of preoperative radiation, preoperative rectal MRI is the most useful test to identify the APR. The APR was visible in 85.9% cases in this study, which is similar to other previous studies (13, 20). Our results showed that the accuracy of using the MRI for determining tumor location relative to the APR was 89.1%, 95.3%, and 93.1% for tumors above, straddle and below the APR, respectively. Corresponding percentages in other studies were 70%, 50%, and 98.2% (20), and 93.5%, 90.0%, and 84.6% (17), respectively, which are similar to our results (17, 20).

The height of APR varies greatly in patients of different sex and body height. Several studies have measured the distance of the APR from the anal verge by intraoperative rigid sigmoidoscopy. In an American study of 50 patients, the mean height of the APR was 9 cm (range: 5.5–13.5 cm) for females, and 9.7 cm (7–16 cm) for males (14). In a Korean study of 46 patients, the mean height of the APR was 8.8 ± 2.2 cm for males and 8.1 ± 1.7 cm for females (12). The position of the APR can also be assessed by rectal MRI. A large study (n=319) using MRI to measure the APR showed that there was a significant difference in the height of the APR between females and males (10.4 ± 1.1 cm vs. 10.0 ± 1.2 cm, P=0.014) (20). Our results showed that the median height of APR measured by MRI was 8.7 cm (4.5–14.3 cm) and positively related with body height, which are consistent with published results (12, 14).

The APR divided rectal cancer into two subtypes: intraperitoneal and extraperitoneal. The local recurrence rate has been found to be much lower in intraperitoneal compared to the extraperitoneal rectal cancer patients (15) and is consistent with our results. The univariate and multivariate analyses of this study showed that only in patients with rectal tumors straddle/below the APR, the radiation group had significant longer LRFS than the no-radiation group, which was consistent with the results of previous works. The Dutch TME trial (4) and Swedish rectal cancer trial (5) demonstrated that local recurrence was reduced significantly in middle and lower rectal cancer, but not in URC. Some studies also suggested that omission of radiation may not jeopardize oncologic outcomes in stage II/III URC (21). In a retrospective study of 547 URC cases, only in high-risk patients (positive lymph node > 6, or tumor deposit) the radiation group had significant longer cancer-specific survival than the no-radiation group (22). Our large retrospective study showed that, for URC with all resection margins negative, there was no significant difference between the radiation group and the no-radiation group in terms of OS, DFS and LRFS.

Rigid sigmoidoscopy is recommended for measuring the height of rectal cancer, but it is performed in only a minority of patients (23) and is not frequently used in China. Instead, flexible colonoscopy, DRE and MRI are generally used instead. The current gold standard for the detection of colorectal cancer is flexible colonoscopy (24). The ESMO guidelines indicate that the difference in measurements obtained by rigid versus flexible colonoscopy is small (25). MRI-based measurements of the distance between inferior tumor margin and the anal verge is a reproducible alternative to rigid sigmoidoscopy (23). However, during rigid sigmoidoscopy, the curve of the rectum is straightened and may lead to an underestimation of the tumor height. During MRI evaluation, however, this distance is measured using the sum of multiple straight lines. In the case of high cancers, several straight lines are often combined to follow the curved line of the rectum, which can result in a longer distance than the actual distance. Our results showed that measurements by different methods highly correlated with each other, although significant differences still existed in many cases (26). Therefore, flexible colonoscopy is an acceptable alternative for rigid sigmoidoscopy if the latter is unavailable.

Our results demonstrated that the APR is a distinct and individualized landmark that can be easily identified by preoperative MRI and intraoperative finding. Patients with rectal cancer above the APR exhibited a lower incidence of local recurrence, and there was no significant difference between the radiation group and the no-radiation group in terms of OS, DFS and LRFS. Tumor location with respect to the APR is an independent predictor of LRFS, while other subdivisions based on a fixed distance measured by DRE, MRI or colonoscopy were not associated with survival outcomes. Therefore, the definition of URC as a rectal tumor above the APR is superior to other definitions, based on a fixed tumor height as measured by MRI and colonoscopy, for selecting patients with stage II/III rectal cancer that should avoid radiation. This definition will not only help us to select suitable cases that should undergo radiation, but also to reduce the incidence of radiation-related toxicity and medical expenses.

There are several limitations in our study. First, this was a retrospective study. In clinical practice, physicians may have preferred to recommend postoperative radiation to high risk patients. Therefore, selection bias is unavoidable. Second, patients with positive resection margin were excluded in this study. Adjuvant radiation might also be needed for some URC patients receiving R1/R2 resection. Third, the included patients did not receive preoperative CRT as recommended by the NCCN and ESMO guidelines due to various reasons. In Asian countries (China, Japan, Korean), postoperative adjuvant CRT or adjuvant chemotherapy is considered the treatment of choice for stage II or III rectal cancer, especially for low risk cases and those with URC (22). Fourth, we do not have the data of tumor location measured by rigid sigmoidoscopy, which is not frequently used in China. The ESMO guidelines indicate that the difference in measurements obtained by rigid versus flexible colonoscopy is small (25). Therefore, the tumor height measured by flexible colonoscopy can, to some extent, replace the measurement by rigid colonoscopy.

## Conclusion

The definition of URC as a rectal tumor above the APR might be better than other definitions based on a numerical tumor height measured by MRI and colonoscopy in selecting patients with stage II/III rectal cancer to avoid adjuvant radiation. The tumor location relative to the APR could be recorded in the preoperative rectal MRI imaging, intraoperative surgical records and postoperative histopathology reports for prognostication and treatment planning (especially adjuvant radiation). However, further prospective RCTs with a larger sample size are required to validate the findings of this study.

## Data Availability Statement

The raw data supporting the conclusions of this article will be made available by the authors, without undue reservation.

## Ethics Statement

The studies involving human participants were reviewed and approved by the ethical committee at Changhai Hospital.

## Author Contributions

FS, LL, and WZ conceptualized the study. XG, BZ, and JL conducted the data curation and wrote the original draft. XG and JL performed the formal analysis. XG acquired the funding. JK, HG, CB, ML, and SZ conducted the investigation. XG developed the methodology. FS and WZ provided the resources. FS, LL, and WZ supervised the study. XG, BZ, JL, JK, HG, CB, ML, SZ, FS, LL, and WZ wrote, reviewed, and edited the manuscript. All authors contributed to the article and approved the submitted version.

## Funding

This study was supported by Shanghai Pujiang Program (#2019PJD052) and the National Natural Science Foundation of China (#81572332).

## Conflict of Interest

The authors declare that the research was conducted in the absence of any commercial or financial relationships that could be construed as a potential conflict of interest.

## Supplementary Material

Supplementary Table 1 The distance between the anal verge and the APR (cm) as measured by MRI.

Supplementary Table 2 Tumor location relative to the APR as determined by MRI and intraoperative findings.

Supplementary Table 3 Univariate and multivariate analyses of risk factors of OS and DFS using a Cox regression model (n = 576).

Supplementary Figure 1 Tumor location relative to the anterior peritoneal reflection (APR) as determined by MRI **(A–C)** and intraoperative palpation and visualization **(D–F)**. The “☆” in the MRI indicates the tumor. The yellow arrow in the MRI indicates the APR. The green curve in intraoperative finding indicates the APR.

Supplementary Figure 2 The relationship between tumor location relative to the APR and overall survival **(A, B)**, postoperative radiation and overall survival **(C, D)** in patients with rectal cancer. APR, anterior peritoneal reflection.

Supplementary Figure 3 The relationship between tumor location relative to the APR and disease free survival **(A, B)**, postoperative radiation and disease free survival **(C, D)** in patients with rectal cancer. APR, anterior peritoneal reflection.

Supplementary Figure 4 Comparison of tumor height measured by digital rectal examination (DRE), MRI and flexible colonoscopy. Scatter plots of discrepancies between the DRE and MRI **(A)**, the DRE and colonoscopy **(B)**, the colonoscopy and MRI **(C)**; Bland-Altman graphs **(D–F)** illustrate the variability between two measurements: mean (central blue line) and 95% confidence intervals (upper and lower red lines).
