# Supplementary material for: Which Definition of Upper Rectal Cancer Is Optimal in Selecting Stage II or III Rectal Cancer Patients to Avoid Postoperative Adjuvant Radiation?
Source: Front Oncol. 2021 Feb 12;10:625459. doi: 10.3389/fonc.2020.625459 (PMC7907590; doi:10.3389/fonc.2020.625459)
Supplement: Supplementary Table 1 — The distance between the anal verge and the APR (cm) as measured by MRI. [file Table_1.docx]

**Supplemental Table 1.** The distance between the anal verge and the APR (cm) as measured by MRI

|  | Cases | Minimum | Maximum | Interquartile range | | | Mean±SD | P (Female vs. male) |
| --- | --- | --- | --- | --- | --- | --- | --- | --- |
|  |  |  |  | 25% | 50% | 75% |  |  |
| Male | 201 | 5.3 | 14.3 | 7.6 | 8.9 | 10.4 | 9.0±1.9 | 0.001 |
| Female | 129 | 4.5 | 12.9 | 7.2 | 8.4 | 9.4 | 8.4±1.5 |  |
| Total | 330 | 4.5 | 14.3 | 7.4 | 8.7 | 9.9 | 8.8±1.8 |  |

APR: anterior peritoneal reflection; SD: standard deviation.
