# Supplementary material for: Which Definition of Upper Rectal Cancer Is Optimal in Selecting Stage II or III Rectal Cancer Patients to Avoid Postoperative Adjuvant Radiation?
Source: Front Oncol. 2021 Feb 12;10:625459. doi: 10.3389/fonc.2020.625459 (PMC7907590; doi:10.3389/fonc.2020.625459)
Supplement: Supplementary Table 2 — Tumor location relative to the APR as determined by MRI and intraoperative findings. [file Table_2.docx]

**Supplemental Table 2.** Tumor location relative to the APR as determined by MRI and intraoperative findings

|  |  | By intraoperative findings | | | |
| --- | --- | --- | --- | --- | --- |
|  |  | Above the APR | Straddle the APR | Below the APR | Total |
| By MRI | Above the APR | 114 | 1 | 2 | 117 |
|  | Straddle the APR | 12 | 82 | 6 | 100 |
|  | Below the APR | 2 | 3 | 108 | 113 |
|  | Total | 128 | 86 | 116 | 330 |
| Accuracy rate |  | 89.1% (114/128) | 95.3% (82/86) | 93.1% (108/116) | 92.1% (304/330) |

APR: anterior peritoneal reflection.
