# Supplementary material for: Which Definition of Upper Rectal Cancer Is Optimal in Selecting Stage II or III Rectal Cancer Patients to Avoid Postoperative Adjuvant Radiation?
Source: Front Oncol. 2021 Feb 12;10:625459. doi: 10.3389/fonc.2020.625459 (PMC7907590; doi:10.3389/fonc.2020.625459)
Supplement: Supplementary Table 3 — Univariate and multivariate analyses of risk factors of OS and DFS using a Cox regression model (n = 576). [file Table_3.docx]

**Supplemental Table 3.** Univariate and multivariate analyses of risk factors of OS and DFS using a Cox regression model (n = 576)

| Parameters | OS | | | | DFS | | | |
| --- | --- | --- | --- | --- | --- | --- | --- | --- |
|  | Univariate | | Multivariate | | Univariate | | Multivariate | |
|  | HR(95%CI) | P | HR(95%CI) | P | HR(95%CI) | P | HR(95%CI) | P |
| Gender (male vs. female) | 0.76(0.36-1.64) | 0.488 |  |  | 1.20(0.78-1.85) | 0.410 |  |  |
| BMI (>=23.59 vs. <23.59kg/m^2^) | 1.09(0.55-2.13) | 0.812 |  |  | 1.01(0.67-1.54) | 0.945 |  |  |
| Diameter (>=4 vs. <4cm) | 1.32(0.64-2.71) | 0.447 |  |  | 1.00(0.66-1.53) | 0.991 |  |  |
| Postoperative pathological TNM stage (Ⅲ vs. Ⅱ) | 6.03(2.12-17.12) | **0.001** | 2.52(0.79-8.02) | 0.117 | 4.65(2.63-8.22) | **<0.001** | 2.86(1.54-5.32) | **0.001** |
| Differentiation (Poor vs. Well/moderate) | 4.37(2.12-9.01) | **<0.001** | 2.74(1.29-5.84) | **0.009** | 1.19(0.63-2.23) | 0.598 |  |  |
| Tumor deposit (Yes vs. No) | 3.52(1.78-6.96) | **<0.001** | 2.12(1.04-4.35) | **0.039** | 4.00(2.63-6.07) | **<0.001** | 2.23(1.41-3.52) | **0.001** |
| Lymphovascular invasion (Yes vs. No) | 5.45(2.75-10.79) | **<0.001** | 2.49(1.17-5.30) | **0.018** | 3.13(2.06-4.74) | **<0.001** | 1.77(1.13-2.76) | **0.012** |
| Perineural invasion (Yes vs. No) | 5.73(2.79-11.76) | **<0.001** | 3.24(1.50-7.00) | **0.003** | 2.33(1.54-3.52) | **<0.001** | 1.32(0.84-2.07) | 0.232 |
| Tumor budding (Yes vs. No) | 1.38(0.58-3.25) | 0.466 |  |  | 1.55(0.94-2.57) | 0.088 |  |  |
| dMMR status (dMMR vs. pMMR) | 1.52(0.36-6.36) | 0.568 |  |  | 0.81(0.26-2.58) | 0.726 |  |  |
| KRAS (Mutant vs. Wild) | 1.45(0.74-2.84) | 0.282 |  |  | 1.36(0.90-2.05) | 0.147 |  |  |
| NRAS (Mutant vs. Wild) | 0.05(0-167.22) | 0.464 |  |  | 2.12(0.93-4.87) | 0.075 |  |  |
| BRAF (Mutant vs. Wild) | 0.05(0-34404) | 0.660 |  |  | 1.73(0.42-7.06) | 0.444 |  |  |
| Postoperative radiation (Yes vs. No) | 1.40(0.68-2.87) | 0.360 |  |  | 1.29(0.81-2.03) | 0.280 |  |  |
| Postoperative chemotherapy (Yes vs. No) | 0.59(0.28-1.27) | 0.181 |  |  | 0.95(0.56-1.61) | 0.843 |  |  |
| CEA (>= 5 vs. <5 ng/ml) | 2.02(1.03-3.95) | **0.041** | 1.81(0.88-3.72) | 0.104 | 1.60(1.06-2.43) | **0.027** | 1.36(0.88-2.11) | 0.168 |
| CA19-9 (>= 37 vs. <37U/ml) | 2.33(1.01-5.35) | **0.046** | 1.64(0.69-3.87) | 0.260 | 1.77(1.02-3.08) | **0.044** | 1.34(0.75-2.39) | 0.319 |
| Tumor location relative to the APR (straddle/below vs. above) | 1.01(0.50-2.01) | 0.983 |  |  | 1.20(0.78-1.85) | 0.416 |  |  |

BMI: body mass index; APR: anterior peritoneal reflection; dMMR: deficient mismatch repair; pMMR: proficient mismatch repair.
