# Supplementary figures and images for: Which Definition of Upper Rectal Cancer Is Optimal in Selecting Stage II or III Rectal Cancer Patients to Avoid Postoperative Adjuvant Radiation?
Source: Front Oncol. 2021 Feb 12;10:625459. doi: 10.3389/fonc.2020.625459 (PMC7907590; doi:10.3389/fonc.2020.625459)

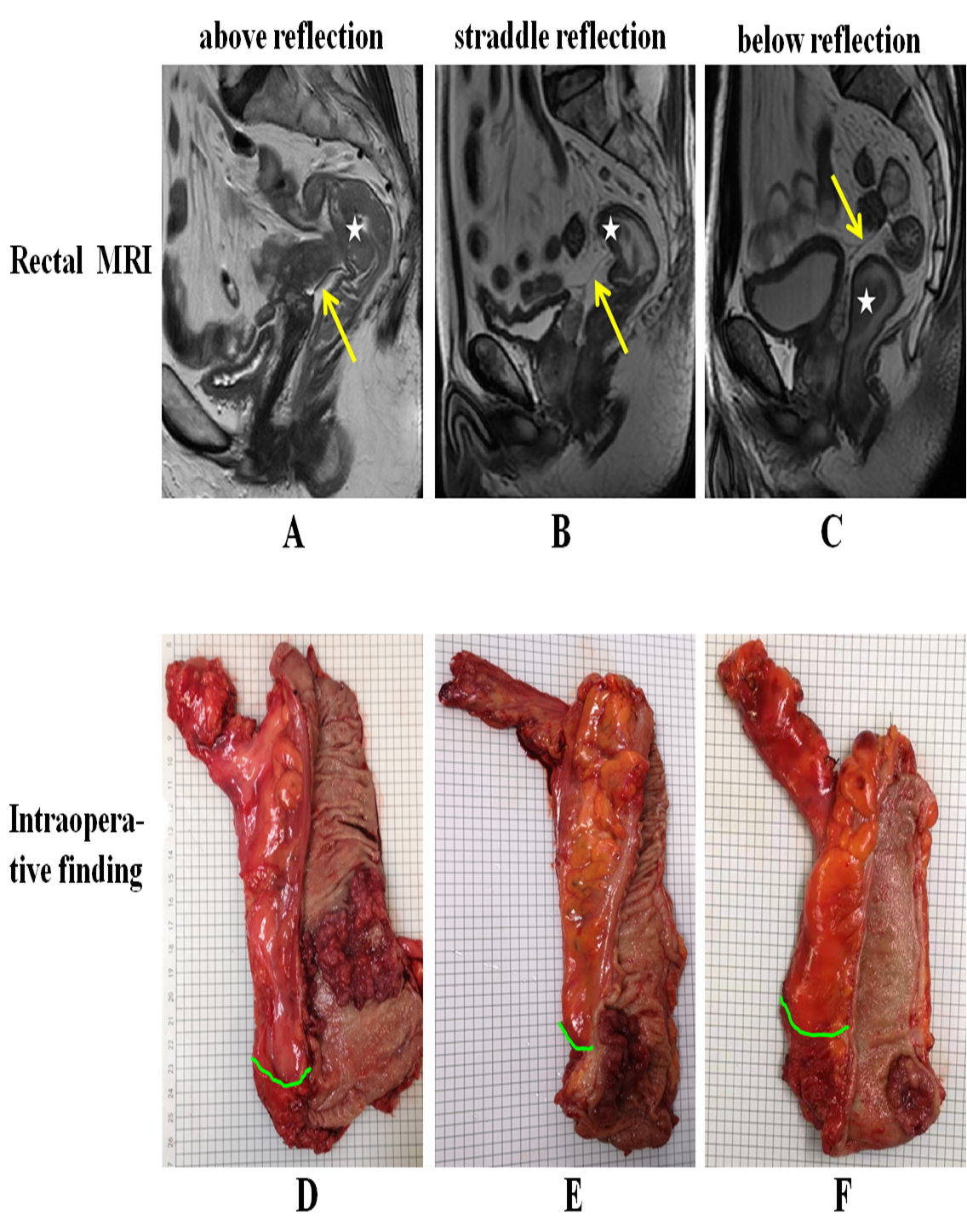

Supplement: Supplementary Figure 1 — Tumor location relative to the anterior peritoneal reflection (APR) as determined by MRI (A–C) and intraoperative palpation and visualization (D–F). The “☆” in the MRI indicates the tumor. The yellow arrow in the MRI indicates the APR. The green curve in intraoperative finding indicates the APR. [file Image_1.tif]

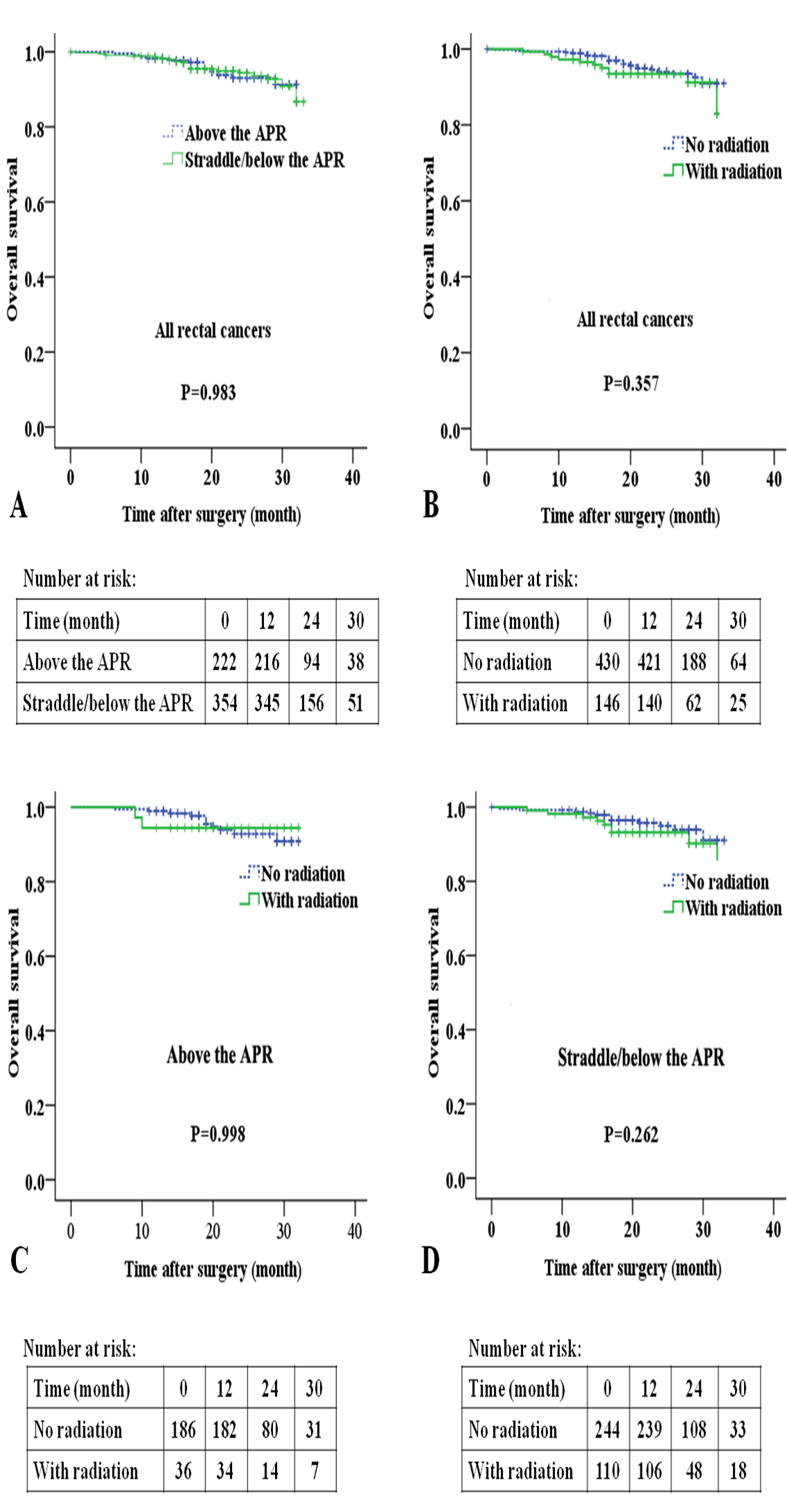

Supplement: Supplementary Figure 2 — The relationship between tumor location relative to the APR and overall survival (A, B), postoperative radiation and overall survival (C, D) in patients with rectal cancer. APR, anterior peritoneal reflection. [file Image_2.tif]

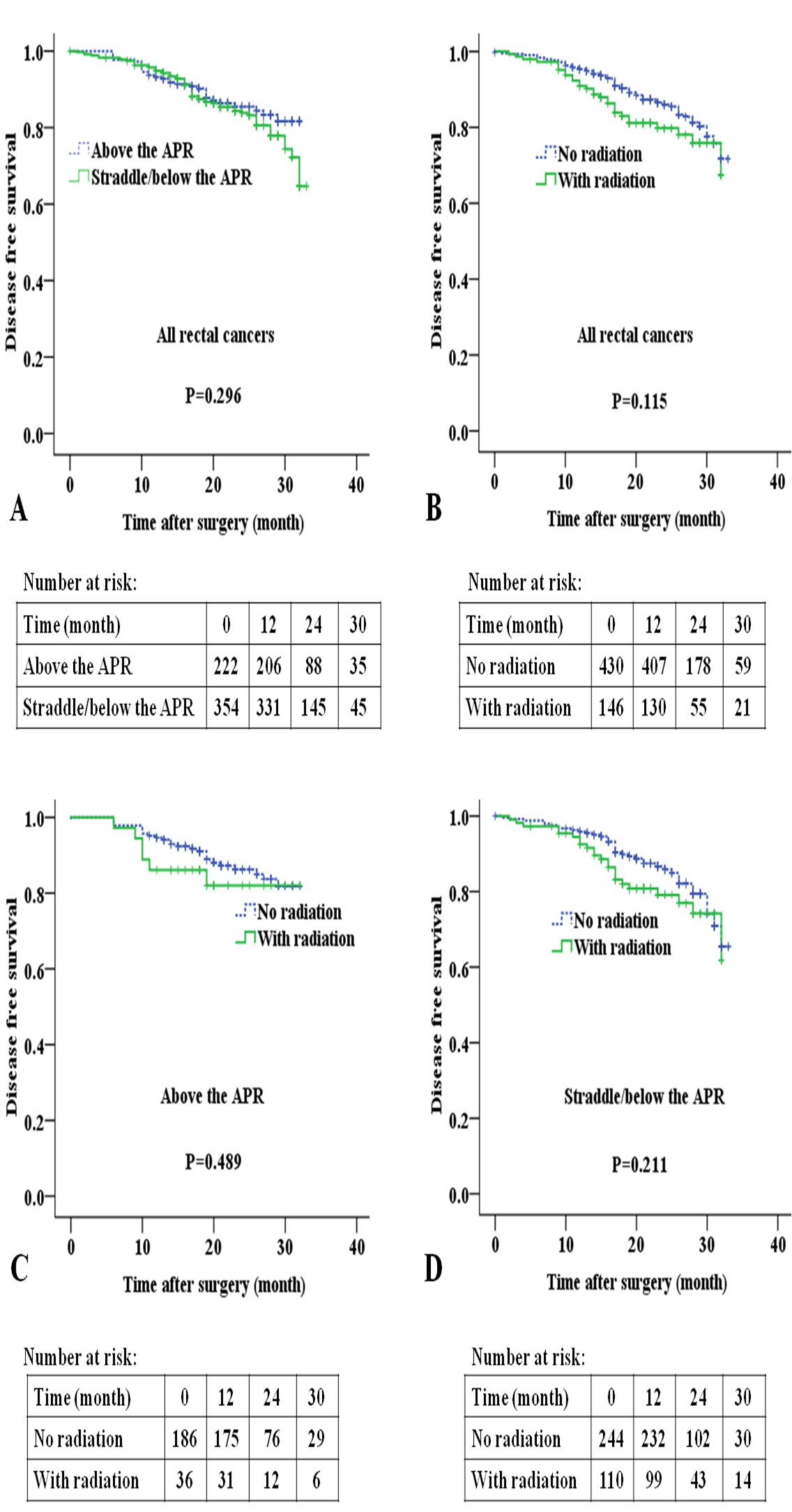

Supplement: Supplementary Figure 3 — The relationship between tumor location relative to the APR and disease free survival (A, B), postoperative radiation and disease free survival (C, D) in patients with rectal cancer. APR, anterior peritoneal reflection. [file Image_3.tif]

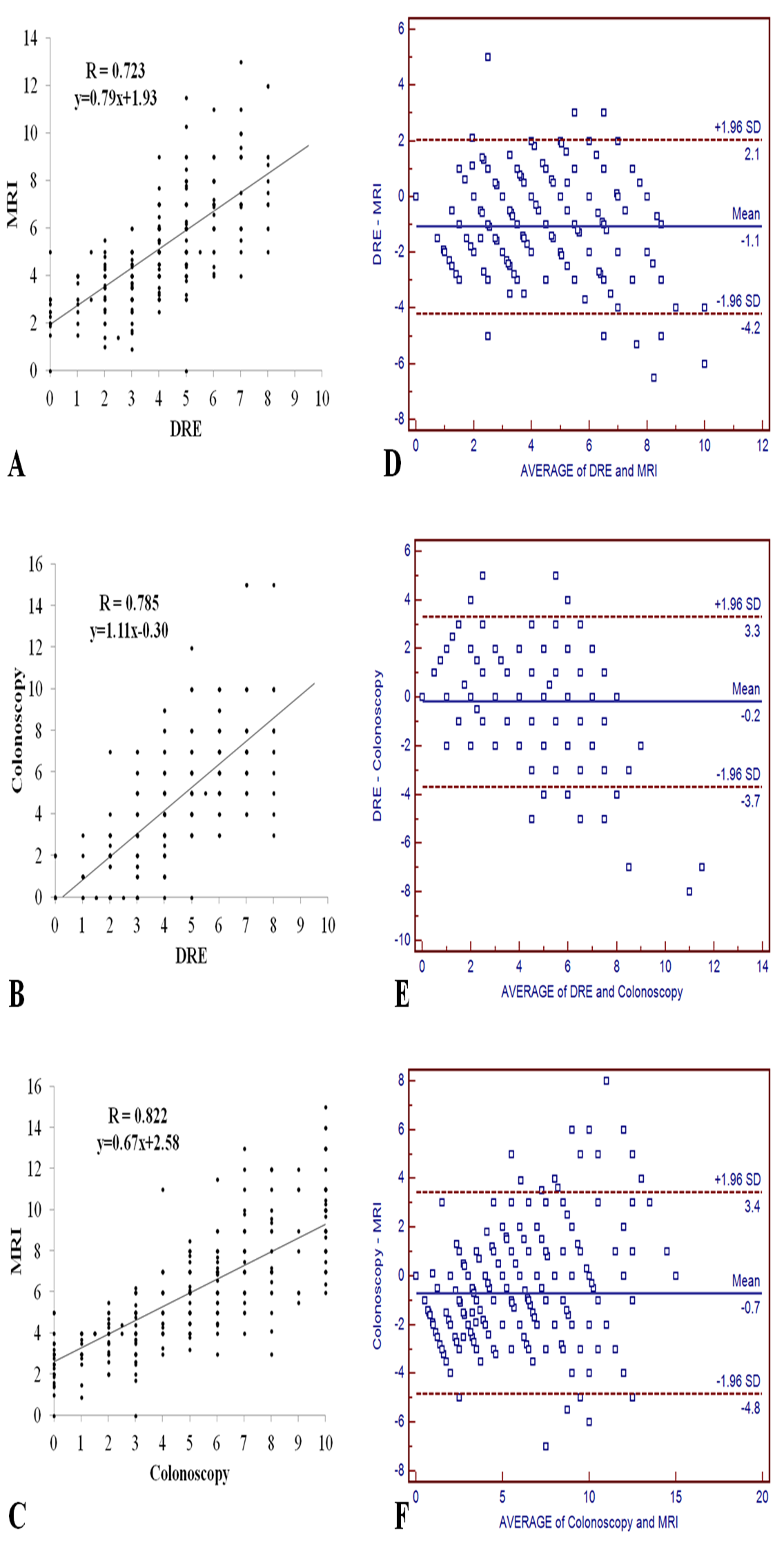

Supplement: Supplementary Figure 4 — Comparison of tumor height measured by digital rectal examination (DRE), MRI and flexible colonoscopy. Scatter plots of discrepancies between the DRE and MRI (A), the DRE and colonoscopy (B), the colonoscopy and MRI (C); Bland-Altman graphs (D–F) illustrate the variability between two measurements: mean (central blue line) and 95% confidence intervals (upper and lower red lines). [file Image_4.tif]
